# Global Transcriptome Profiling Analysis of Inhibitory Effects of Paclobutrazol on Leaf Growth in Lily (*Lilium* Longiflorum-Asiatic Hybrid)

**DOI:** 10.3389/fpls.2016.00491

**Published:** 2016-04-19

**Authors:** Xiaopei Zhu, Min Chai, Yang Li, Meiyu Sun, Jinzheng Zhang, Guofeng Sun, Chuangdao Jiang, Lei Shi

**Affiliations:** ^1^Key Laboratory of Plant Resources and Beijing Botanical Garden, Institute of Botany, Chinese Academy of SciencesBeijing, China; ^2^University of Chinese Academy of SciencesBeijing, China

**Keywords:** lily, dwarfism, RNA-seq, GA, cell division, cell expansion

## Abstract

As a popular ornamental flower, potted lily is an important object of lily breeding. Paclobutrazol, a chemical growth retardation compound, is often used to dwarf plant in producing potted lilies. However, in recent years, the plants with inherited dwarf traits by using genetic engineer breeding technology are being developed. The studies on molecular basis of lily dwarfism will offer some target genes which have profound dwarf effect for genetic engineer breeding. Here, we confirmed that paclobutrazol inhibited plant height and leaf size in *Lilium* Longiflorum-Asiatic hybrid, and then RNA-Seq technique was employed to analyze gene transcripts of *Lilium* Longiflorum-Asiatic hybrid leaves by paclobutrazol treatment in order to get a deeper insight into dwarfism mechanism of lily. Approximately 38.6 Gb data was obtained and assemble into 53,681 unigenes. Annotation, pathways, functional classification and phylogenetic classification of these data were analyzed based on Nr, Nt, Swiss-Prot, KEGG, COG, and GO databases. 2704 differentially expressed genes were screened by comparing paclobutrazol-treated samples with untreated samples and quantitative real-time PCR was performed to validate expression profiles. By analyzing dynamic changes of differentially expressed genes, nine metabolic pathways and signal transduction pathways were significantly enriched and many potentially interesting genes were identified that encoded putative regulators or key components of cell division, cell expansion, GA metabolism and signaling transduction and these genes were highlighted to reveal their importance in regulation of plant size. These results will provide a better understanding of the molecular mechanism on lily dwarfism and some potential genes related to lily organ size, which will lay the foundation for molecular breeding of potted lilies. These transcriptome data will also serve as valuable public genomic resources for other genetic research in lily.

## Introduction

Lily is a perennial ornamental bulb plant. Lily has huge commercial value in the floriculture industry. It can be divided into three classes based on distinct uses: cut flowers, potted plants and landscape plants (Kole, 2011). In the recent years, the potted lilies have become more and more popular in the market. However, tall stem is a major limiting factor when producing potted lilies. Most lily cultivars can reach up to 1 m and must be dwarfed for aesthetic value and less volume during transportation (Miller, 1992). At present, a primary means of dwarfing lily height is to use plant growth regulators (PGRs). Paclobutrazol (PBZ) is one of PGRs and has been practically used in agronomic and horticultural crops (Lever et al., 1982). One remarkable effect of PBZ is to reduce the plant height, meanwhile, it also induces a variety of morphological and physiological responses in leaves including reducing leaf area (Nair et al., 2009; Cohen et al., 2013), increasing chlorophyll contents (Sunitha et al., 2004), improving level of antioxidants (Srivastav et al., 2010) and enhancing resistance to abiotic stresses (Ozmen et al., 2003; Zhu et al., 2004; Lin et al., 2006) and so on.

In addition to application of PGRs, the plants with inherited dwarf traits by using genetic engineer breeding technology are being developed during recent years. Transgenic and compact geranium was obtained through overexpression of an aspartic protease gene, but the number of petals in the flowers was reduced (Chabannes et al., 2009). The *chrysanthemum* (Petty et al., 2003) and *petunia* (Tanaka et al., 2005) became dwarf after introduction of a *gai* (gibberellic acid insensitive) gene. However, currently there are no dwarf varieties by genetic engineer breeding on the marketplace. An understanding of the molecular basis of plant dwarfism offers the prospect of isolation of target genes which may be used in genetic engineer breeding to improve plant architecture. According to morphological studies, dwarf plants rely on the reduction of cell number and/or size, which are governed by cell expansion and cell division processes (González and Inzé, 2015). Cell expansion includes cytoplasmic growth, turgor driven cell-wall extension and rigidification of the cell wall (Passardi et al., 2004; Humphrey et al., 2007; Sablowski and Dornelas, 2014). The cell division process comprises a continuous cycle of pre-DNA synthesis phase (G1 phase), DNA synthesis phase (S phase), DNA repair phase (G2 phase), and mitotic phase (M phase) (Inzé and De Veylder, 2006). A large number of genes involved in these processes have been identified, and their overexpression or down-expression affect plant growth and size. Overexpression of an extensin gene which involved in rigidification of the cell wall results in dwarf phenotype in *Arabidopsis* (Roberts and Shirsat, 2006). Cyclin-dependent kinases (CDKs) trigger G1-to-S and G2-to-M transition in the cell division and inhibition of the expression of the *cdc2b* gene encoding B-type CDK results in short-hypocotyl and open-cotyledon phenotypes in *Arabidopsis* (Yoshizumi et al., 1999).

Based on studies of signaling regulation on plant dwarfism, one major factor is the alteration of gibberellin (GA) metabolism or disruption of the GA signal transduction. Introduction of semi-dwarf trait in rice and wheat lead to “Green Revolution” that improved crop yield (Hargrove and Cabanilla, 1979; Perovic et al., 2008). Analysis of molecular genetics shows that the semi-dwarf phenotype of rice is due to the mutation of a GA metabolic gene *GA20ox* (Monna et al., 2002; Sasaki et al., 2002) and reduced height of wheat is due to the mutation of a GA responsive gene *Rht1* encoded DELLA protein (Peng et al., 1999). GA metabolism contains GA biosynthesis and deactivation. The GA biosynthesis involves in six enzymes, namely *ent*-copalyldiphosphate synthase (CPS), *ent*-kaurene synthase (KS), *ent*-kaurene oxidase (KO), *ent*-kaurenoic acid oxidases (KAO), GA20-oxidases (GA20ox), and GA3-oxidases (GA3ox), while GA2-oxidases (GA2ox) are the important enzymes for GA deactivation (Hedden and Phillips, 2000). Overexpression or repression of genes encoding these enzymes could lead to the alteration of GA levels and thereby result in dwarf or tall phenotypes (Lange and Lange, 2006). In GA signal transduction, bioactive GAs bind to the receptor GIBBERELLIN INSENSITIVE DWARF 1 (GID1), forming GAs-GID1 complexes to bind to DELLA proteins which are negative regulators of GA response, and then the GID2, one part of the E3 ubiquitin ligase complex, mediates the degradation of DELLA proteins by the 26S proteasome (Sasaki et al., 2003; Griffiths et al., 2006). If mutation of these components of GA signal transduction occurs, the transmission of GA signals is disrupted, resulting in dwarf or tall phenotypes (Schwechheimer and Willige, 2009).

Lilies have very huge genomes and lack the genomic information, and relatively little is known about molecular basis of dwarfism in this species. RNA-Seq technology is recently developed approach that allows the entire transcriptome to be surveyed in a high-throughput and quantitative manner (Wang et al., 2009). This method is capable of measuring transcriptome composition and detecting digital gene expression levels at the genome scale, and it is not dependant on the existing genomic sequence, thus RNA-Seq technology has provided a comprehensive and efficient way to analyze transcriptome for non-model organisms without genomic sequences (Wang et al., 2009). In this study, we used *Lilium* Longiflorum-Asiatic hybrid (*L*. LA hybrid), which was appropriate for potted plant or garden cultivation, as the experimental materials. We performed *de novo* transcriptome sequencing of PBZ-treated and untreated lily leaves, and then we tried to analyze these data to understand molecular processes of dwarfism in lily. This dataset will serve as valuable resources for novel gene discovery, genomics, and functional genomics in lily. Furthermore, studying molecular mechanism of dwarfism will be valuable in efforts to alter plant type by genetic breeding in lily.

## Materials and methods

### Plant materials and PBZ solution spraying

The *L*. LA hybrid “Ceb Dazzle” were obtained from a commercial source and used as experimental materials. Precooled lily bulbs (12 cm circumference) were potted in the greenhouse of the Institute of Botany, the Chinese Academy of Sciences, under the following growth conditions: 60% relative humidity, day/night temperatures, 20–25°C/15–18°C, with watering every 5 days. The soil was a 2:1(V: V) mixture of peat moss and sand with APEX fertilizer (14-14-14). When the seedlings were 10 cm in height (about 30 days), they were randomly assigned into four groups and were applied by PBZ solution with concentration of 0 (water control), 100, 300, 500 mg L^−1^ using foliar spray, respectively.

### Leaf size measurement and epidermal cell observation after PBZ treatment

The first and second leaves of 10 cm tall plants were tagged prior to PBZ treatment and collected at 10 days after PBZ treatment. The leaf length and width were measured with a digital vernier caliper, and the leaf area was measured using a LICOR photoelectric area meter (model L1-3100, Lincoln, USA). For the evaluation of epidermal cells, the leaves were fixed in FAA solution (50% ethanol: acetic acid: formaldehyde = 18: 1: 1) for at least 24 h, and then dehydrated in a graded ethanol series (70–100%), followed by exchange in a graded isoamyl acetate series. The samples were dried with a CO_2_ critical point drier and coated with gold, and they were then studied with a scanning electron microscope (SEM, model S-4800 FESEM, Japan).

### RNA isolation and cDNA library construction for illumina sequencing

Mixed leaves of three individuals were separately collected at 3, 24, and 72 h after PBZ treatment and from untreated control, immediately frozen in liquid nitrogen and stored at −80°C until use. Two replicates were performed. Total RNA of each sample was obtained using an RNAprep Pure Plant kit (TIANGEN Biotech, Beijing, China) according to the manufacturer's instructions. The quality of RNA was determined using the NanoDrop 1000 spectrophotometer and an Agilent 2100 bioanalyzer (Agilent Technologies, Santa Clara, USA), and the RNA integrity number (RIN) value of all sequencing samples was more than 8.0. The cDNA libraries were constructed as described below. Total RNA of each sample was treated with DNase I, and poly (A) mRNA was then isolated with oligo-dT beads, followed by reverse-transcription into first-strand cDNA using reverse transcriptase. Second-strand cDNA was synthesized using DNA polymerase I and RNaseH, and then ligated with an adaptor or index adaptor using T4 DNA ligase. Adaptor-ligated fragments were separated and excised by agarose gel electrophoresis. PCR was performed to selectively enrich and amplify the cDNA fragments. Finally, the cDNA libraries were sequenced using an Illumina HiSeq 2000 at BGI Tech Solutions Co., Ltd. (BGI Tech, Shenzhen, China).

### *De novo* assembly and functional annotation

Adaptor sequences, reads with unknown sequences and low quality reads were first removed from data produced from sequencing machines, and the clean reads were then assembled using Trinity software (http://trinityrnaseq.sourceforge.net; Grabherr et al., 2011). Data obtained for each sample were separately assembled, and the assembly sequences were called unigenes. The unigenes from all samples were further subjected to sequence splicing and redundancy removal with sequence clustering software to acquire non-redundant unigenes as long as possible. Some unigenes in which the similarity was more than 70% were designated by the prefix CL, the others were singletons, in which the prefix was unigene. These unigene sequences were aligned and annotated to protein databases like Nr, Swiss-Prot, COG (Tatusov et al., 2001), GO (Conesa et al., 2005), KEGG (Kanehisa et al., 2012), and nucleotide database Nt with a threshold of *E* < 10^−5^. The best aligning results were used to decide sequence direction of unigenes. If results from different databases conflicted with each other, a priority order of Nr, Swiss-Prot, KEGG and COG should be followed when deciding sequence direction of unigenes.

### Analysis of differentially expressed genes (DEGs)

Expression of the unigenes was calculated using the FPKM (Fragments Per Kb per Million reads) method, which eliminated the influence of lengths and sequencing discrepancies of different genes on the gene expression calculations. The FPKM between the biological replications was analyzed using Pearson correlation, and the 0 value was replaced by 0.01 to calculate the fold change. According to the correlation results, DEGs were selected on condition of *Q* ≥ 0.8 and an absolute value of log_2_ratio (treatment/control) ≥1 based on the NOISeq method (Tarazona et al., 2011).

### Quantitative real-time PCR (qRT-PCR) validation

Total RNA was separately extracted from leaves at 3, 24, and 72 h after PBZ treatment and leaves from untreated control as described earlier. First-strand cDNA synthesis was performed using the PrimeScript® RT reagent kit (Takara Biotechnology, Dalian, China) according to the manufacturer's instructions. The primers of 12 unigenes were designed using Primer Express software (version 3.0.1) and listed in Table S1. The *actin* gene was used as an internal reference gene. qRT-PCR was performed using a 7500 Fast Real-Time PCR System using Power SYBR Green PCR Master Mix (Applied Biosystems, Carlsbad, USA). For each sample, reactions were performed in triplicate, and relative mRNA levels were calculated using the 2^−ΔΔCt^ method (Livak and Schmittgen, 2001).

## Results

### PBZ reduced plant height and leaf area

**Figure 1:**
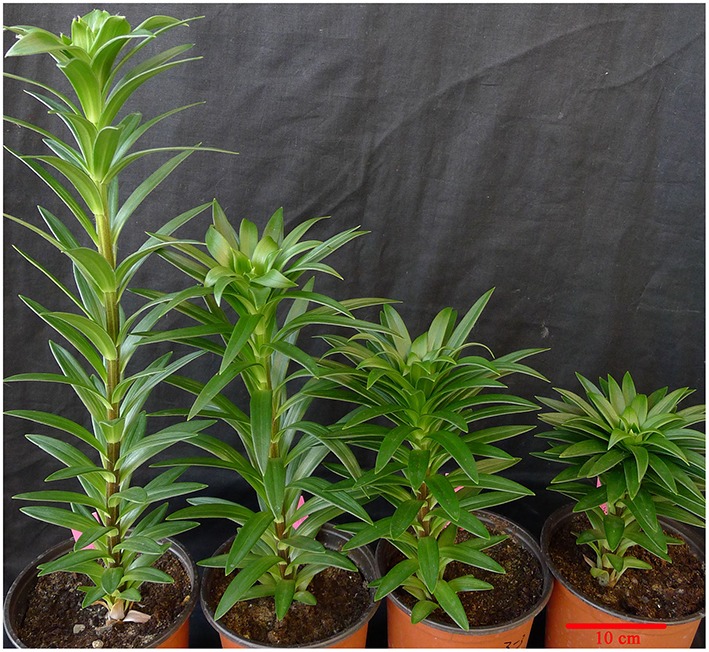
**Dwarfing trait in *L*. LA hybrid induced by PBZ with different concentrations**. From left to right: CK (water control), 100 mg L^−1^ PBZ-treated plant, 300 mg L^−1^ PBZ-treated plant, 500 mg L^−1^ PBZ-treated plant. Scale bar: 10 cm.

**Figure 2:**
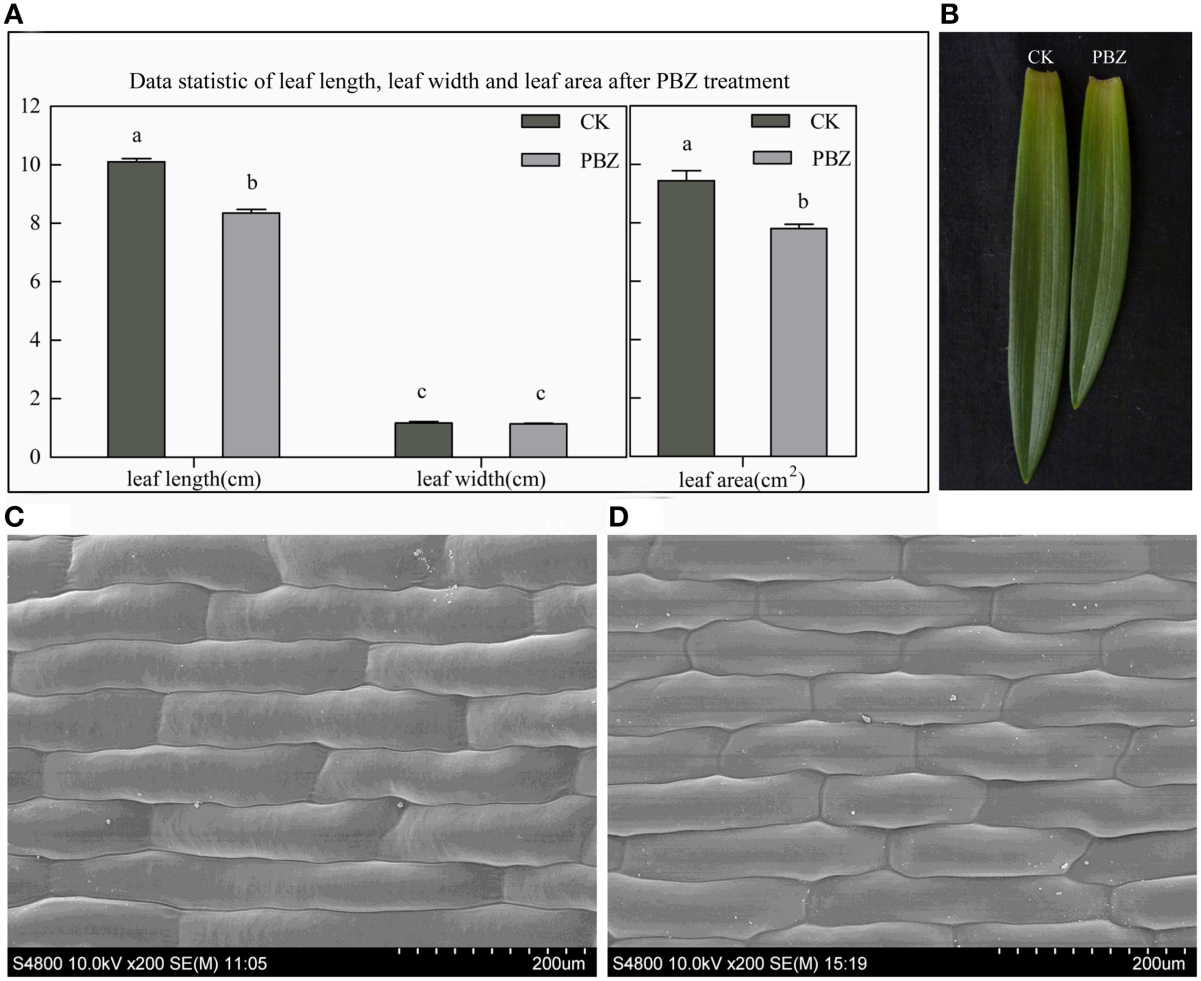
**Morphological analysis of PBZ-treated leaves and leaf cell compared with controls (CK). (A)** Leaf length, leaf width and leaf area measurements. Values are presented as the mean ± standard deviation (SD) of five samples in each group. Values sharing different superscript letters differ significantly (*P* ≤ 0.05). **(B)** Photograph of the individual leaves from PBZ-treated samples and CK. **(C,D)** Morphology of epidermal cells from the middle area of a half leaf next to the midvein by SEM; **C**, controls; **D**, PBZ-treated; the scale is 200 μm.

Plant height was shorter for all PBZ-treated plants compared with control plants and was suppressed more and more severely as PBZ concentration increased (Figure 1). To study acting mechanism of PBZ on lily, the most severely dwarf group applied by 500 mg L^−1^ PBZ solution and the control group were used as following research. The leaf shape between the two groups was firstly observed. The leaf length, leaf width and leaf area were measured. The results showed that PBZ significantly decreased leaf area and leaf length but had no effect on leaf width in lily (Figures 2A,B). We further observed the upper epidermal cells of leaves and found that the cell length of lily leaves was decreased but cell width was not affected by PBZ treatment (Figures 2C,D). These results indicated that PBZ may inhibit leaf length by decreasing cell length in lily.

### Sequencing and *De novo* assemably of *L*. LA hybrid transcriptome

The eight cDNA samples were prepared from PBZ-treated groups at 3, 24, 72 h and control group and sequenced using the Illumina HiSeq 2000. After stringent quality check and data cleaning, 4.61–4.99 Gb clean nucleotides were obtained for each sample, totaling 38.59 Gb nucleotides for all samples (Table 1). The Q20 percentage (sequencing error rate < 1%) and GC percentage were 96.60–97.54% and 52.09–52.77%, respectively (Table S2). On the basis of the high-quality reads, 90,995–97,050 contigs were firstly assembled with an average length of 311–321 bp for each sample (Table S2). Then these contigs in each sample were separately assembled in 45,399–50,326 unigenes with an average length of 581–606 bp (Table 1). To maximize the number and length of genes, the eight groups of assembly data were mixed and assembled again. Finally, a total of 53,681 unigenes, of which 25,773 were clusters and 27,908 singletons, were obtained with an average length of 849 bp (Table 1).

**Table 1:** **Sequence statistics of the *L*. LA hybrid transcriptome**.

**Sample**	**Total clean nucleotides (nt)**	**Number of unigenes**	**Mean length of unigenes (nt)**	**Distinct clusters**	**Distinct singletons**	**GenBank accession number**
CK-1	4,950,814,860	46,625	589	14,281	32,344	SRR3233709
CK-2	4,632,250,500	46,483	586	14,085	32,398	SRR3233711
T3-1	4,612,069,440	45,399	600	13,911	31,488	SRR3233712
T3-2	4,863,475,260	46,211	600	14,356	31,855	SRR3233713
T24-1	4,659,464,340	47,682	586	14,305	33,377	SRR3233714
T24-2	4,986,718,740	50,326	581	15,389	34,937	SRR3233715
T72-1	4,922,844,840	46,452	606	14,455	31,997	SRR3233716
T72-2	4,967,284,860	49,843	582	15,187	34,656	SRR3233717
All	38,594,922,840	53,681	849	25,773	27,908	

*CK-1 and CK-2, two biological replicates of control samples; T3-1 and T3-2, two biological replicates of PBZ-treated samples at 3 h; T24-1 and T24-2, two biological replicates of PBZ-treated samples at 24 h; T72-1, and T72-2, two biological replicates of PBZ-treated samples at 72 h*.

**Figure 3:**
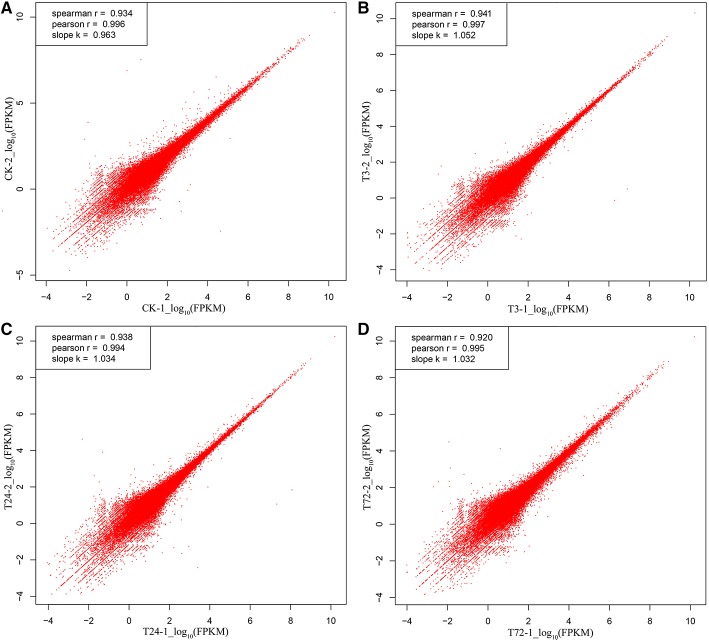
**Pearson correlation analysis of the two replicates in each group**. The vertical and horizontal axes represent the scale of the expression levels for all unigenes [log_10_RPKM (number of reads per kilobase per million clean reads)]. **(A)** control samples (CK-1 and CK-2); **(B)** PBZ-treated samples in 3 h (T3-1 and T3-2); **(C)** PBZ-treated samples at 24 h (T24-1 and T24-2); **(D)** PBZ-treated samples at 72 h (T72-1 and T72-2).

To evaluate biological variability between individuals, Pearson correlations were determined by expression levels of unigenes among the biological replicates. The Pearson coefficient from controls (CK-1 and CK-2), PBZ-treated 3 h (T3-1 and T3-2), PBZ-treated 24 h (T24-1 and T24-2), and PBZ-treated 72 h (T72-1 and T72-2) was 0.996, 0.997, 0.994, and 0.995, respectively. This result indicated consistency between the biological replicates (Figure 3).

### Functional annotation

**Figure 4:**
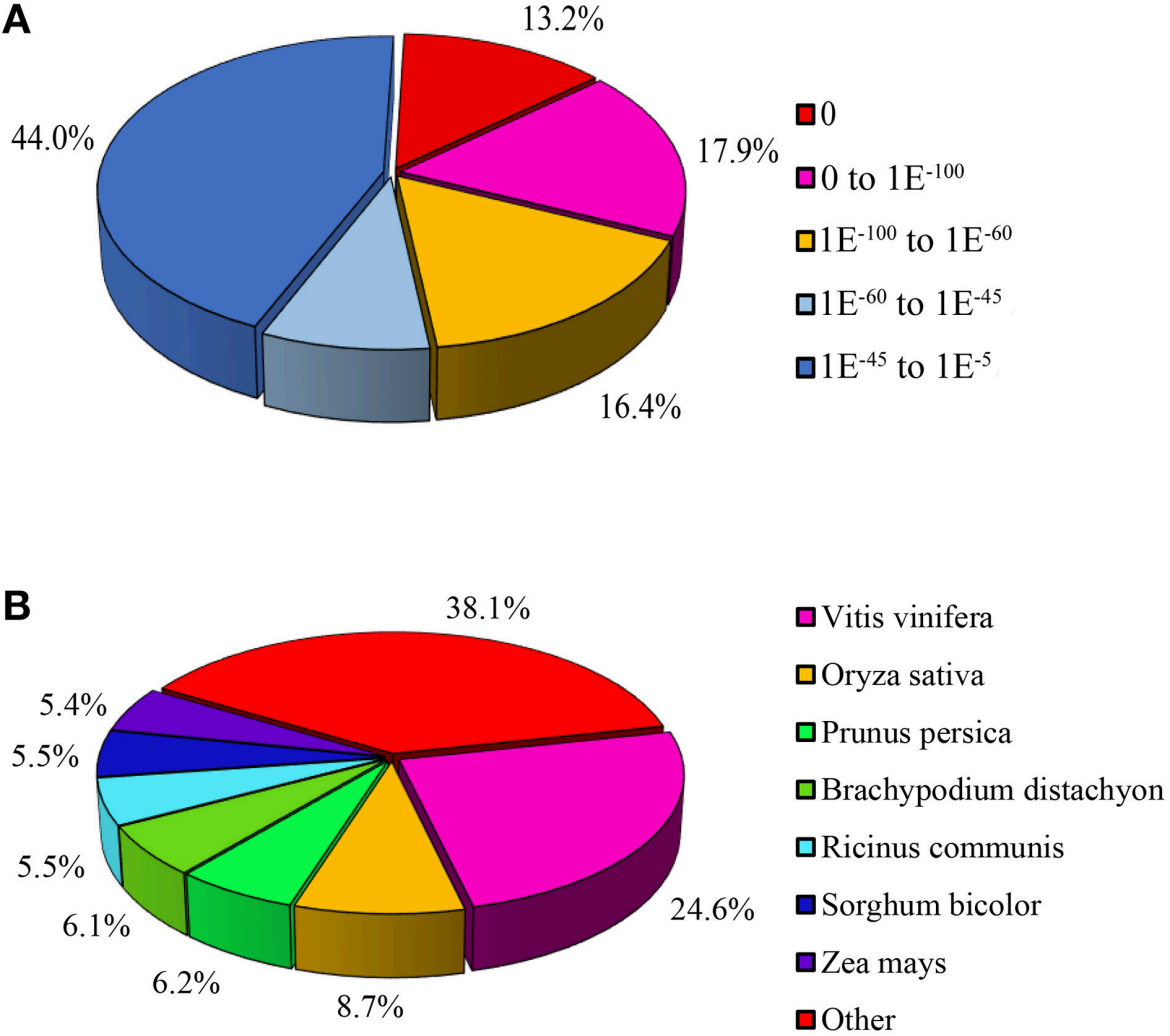
**Characteristics of homology search of unigenes aligned by BLASTx to the nr database. (A)** *E*-value distribution of unigenes annotated in the nr database. **(B)** Species distribution of the first BLAST hits for each sequence with a cut-off *E*-value of 1.0E^−5^.

All unigenes were aligned to five protein databases including Nr, Swiss-Prot, COG, GO, KEGG and one Nt nucleotide database. A total of 38,507 unigenes were annotated, which contained 37,776 (70.37%) unigenes identified from Nr, 27,087 (50.46%) from Nt, 27,316 (50.86%) from Swiss-Prot, 17,201 (32.04%) from COG, 28,014 (52.19%) from GO, 25,092 (46.74%) from KEGG (Table 2). E-value indicated the extent of sequence homology, and E-value is smaller, the sequence homology is higher. For E-value distribution of unigenes blastx hits in the Nr database which had the largest number of annotated unigenes, 44% homolog sequences ranged between 1E^−5^ and 1E^−45^, while 56% sequences had a threshold E-value less than 1E^−45^ that showed strong homology (Figure 4A). Species distribution of the first blastx hits of each unigene in the Nr database indicated that *Vitis vinifera* provided the best blastx matches with 24.6% unigenes in *L*. LA hybrid, and *Oryza sativa* was the second closest species, which had 8.7% homology with *L*. LA hybrid (Figure 4B).

**Table 2:** **Annotation of unigene sequences**.

**Database**	**Number of annotated unigenes**	**Percentage (%)**
Nr	37,776	70.37
Nt	27,087	50.46
Swiss-Prot	27,316	50.86
COG	17,201	32.04
GO	28,014	52.19
KEGG	25,092	46.74
All	38,507	71.73

*Nr, non-redundant NCBI protein database; Nt, non-redundant NCBI nucleotide database; Swiss-Prot, Swiss-Prot protein database; COG, Clusters of Orthologous Groups; GO, Gene Ontology; KEGG, Kyoto Encyclopedia of Genes and Genomes Pathway*.

**Figure 5:**
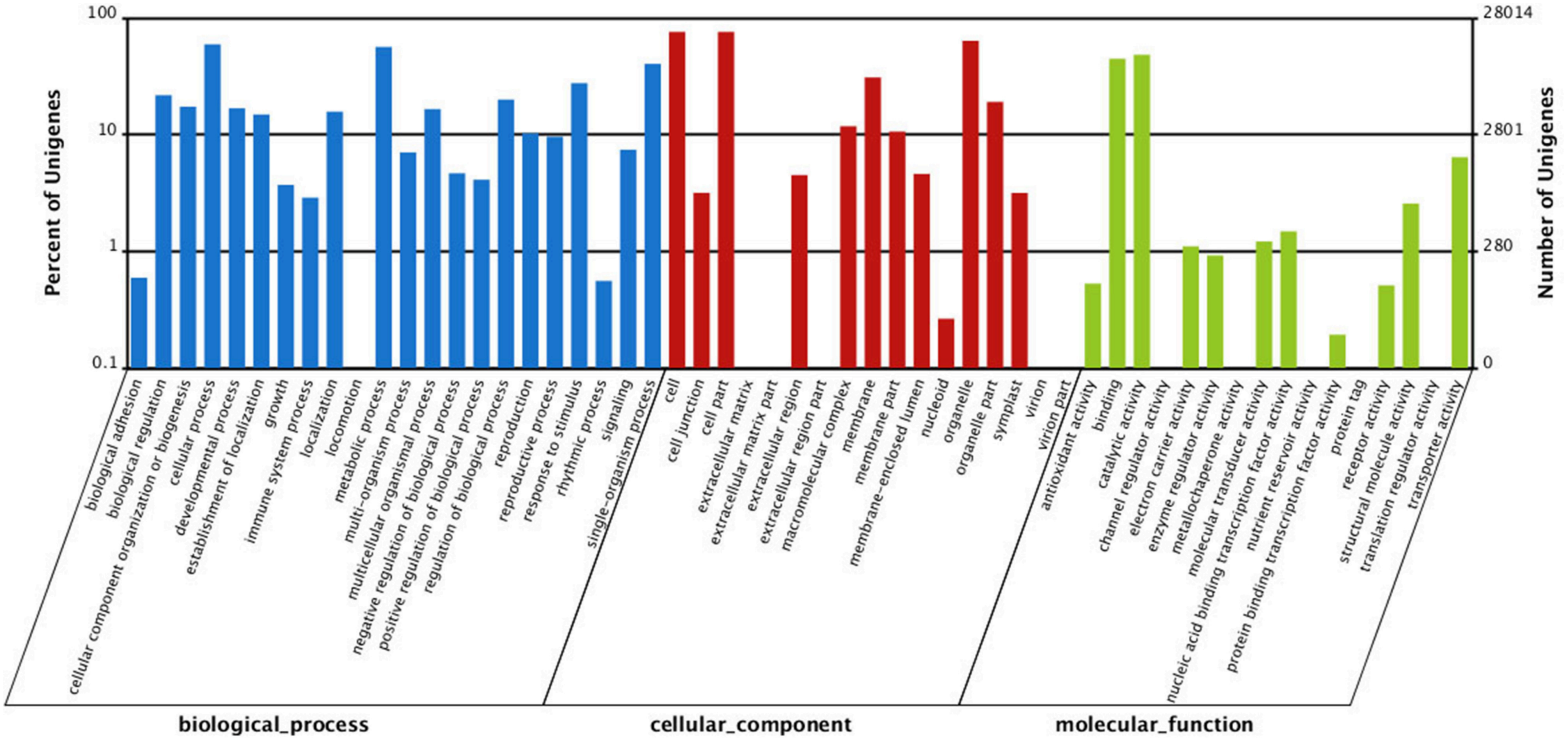
**Gene Ontology (GO) classification of all unigenes**. They are classified into three GO categories: biological process (blue), cellular component (red), and molecular function (green).

**Figure 6:**
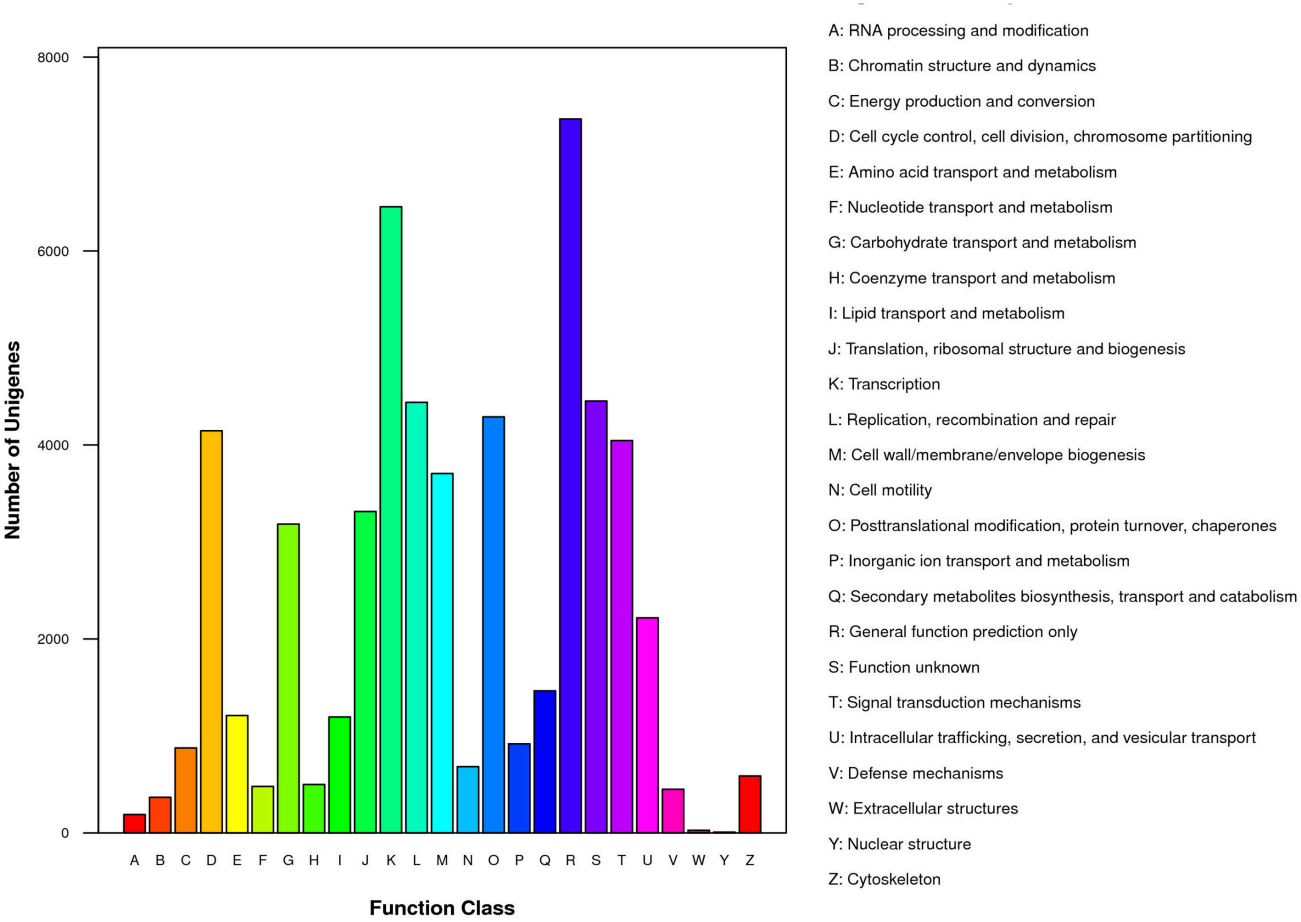
**COG function classification of all unigenes**.

To gain insight into functions of the annotated genes from the macro level, GO functional classification was performed. A total of 55 GO terms were categorized into three domains: biological process, cellular component and molecular function (Figure 5). The terms of “cellular process,” “metabolic process,” and “single-organism process”; “cell,” “cell part,” and “organelle”; “binding” and “catalytic activity” were the most representative of biological process, cellular component, and molecular function, respectively (Figure 5). COG analysis was performed to predict phylogenetic classification. In total, 17,201 unigenes were matched and grouped into 25 functional classes. The clusters for “General function prediction only” (7361) and “Transcription” (6456) were the two largest groups (Figure 6). KEGG enrichment of the unigenes was carried out to analyze pathways involved in *L*. LA hybrid transcriptome and 128 pathways including 25,092 unigenes (46.74%) were identified (Table S3). The “metabolic pathways” had the greatest members (7532, 30.02%), followed by “endocytosis” (3038, 12.11%), “glycerophospholipid metabolism” (2908, 11.59%), “ether lipid metabolism,” (2756, 10.98%) and “biosynthesis of secondary metabolites” (2526, 10.07%).

### Identification of DEGs

**Figure 7:**
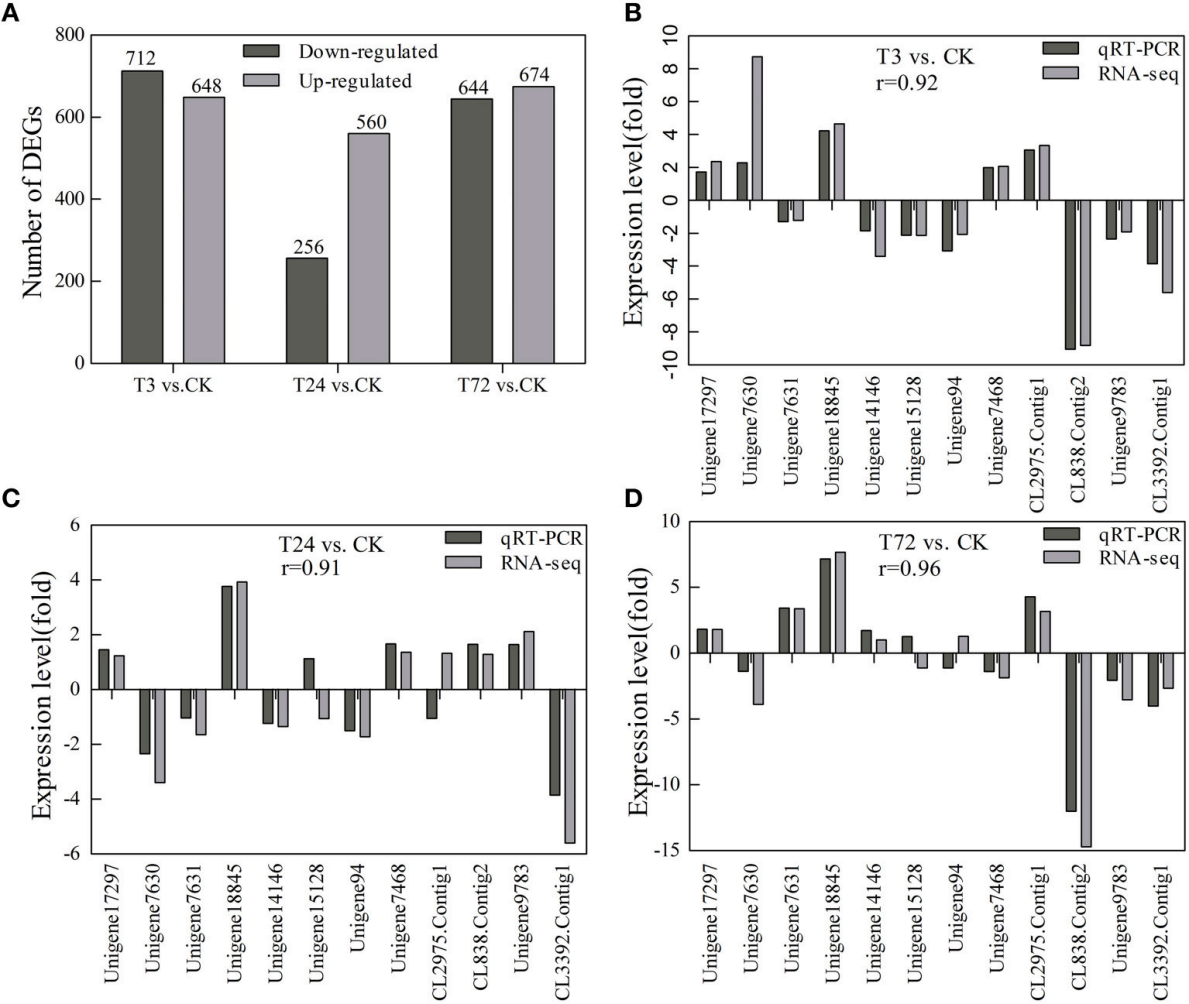
**Statistics of the DEG numbers and verification of expression profiles of 12 selected DEGs using qRT-PCR**. CK, controls; T3, PBZ-treated samples at 3 h; T24, PBZ-treated samples at 24 h; T72, PBZ-treated samples at 72 h. **(A)** The DEG numbers are counted. The black columns showed the upregulated DEGs and the gray columns showed the downregulated DEGs compared with controls. **(B–D)** Close correlations (*r* = 0.92, 0.91, and 0.96) were observed between relative expression levels measured with RNA-Seq and qRT-PCR. The x-axis represented unigene ID, and the y-axis showed expression levels (fold difference).

With Q-value ≥ 0.8 and an absolute log_2_ratio (treatment/control) ≥1 based on the NOISeq method, a total of 2704 genes were found to be differentially expressed among the three PBZ-treated samples compared with control group. Among these DEGs, 648 genes were quickly induced by PBZ, while 712 genes were downregulated at 3 h. At 24 h, 815 genes were found, of which 560 genes were upregulated and 256 genes were downregulated. At 72 h, 674 genes were upregulated and 644 genes were downregulated (Figure 7A). These results indicated that both up- and down-regulation of gene expression occurred, and the transcript abundance of genes changed dynamically over time of PBZ treatment.

To validate DEGs expression profiling obtained by RNA-seq, 12 genes with higher or lower expression levels were selected for qRT-PCR analysis. They included five genes involved in GA metabolism and signal transduction pathway (unigene17297, unigene7630, unigene7631, unigene18845, and unigene14146, encoding KAO, GA20ox, GA20ox, GA20ox, and DELLA, respectively), three genes involved in cell division and cell expansion (unigene15128, unigene94, and unigene7468, encoding CDKB2, expansin and xyloglucan endotransglycolase/hydrolase XTH, respectively), three genes involved in protein processing in endoplasmic reticulum (CL838.Contig2, unigene9783, and CL3392.Contig1, encoding luminal-binding protein, heat shock protein 70 and heat shock protein 17) and one gene encoding high mobility group B protein (CL2975.Contig1). For all of the genes, the results obtained from the RT-PCR analysis were identical to those from RNA-Seq (Figures 7B–D).

### KEGG enrichment analysis of DEGs

To further understand on how PBZ regulates plant growth at the molecular level in *L*. LA hybrid, we mapped the DEGs to the terms in the KEGG database, and significantly enriched metabolic pathways or signal transduction pathways were identified by comparing with the whole genome background. In this study, there were 471 DEGs in T3 sample, 234 DEGs in T24 sample and 462 DEGs in T72 sample mapped to KEGG pathways. Totally, nine pathways were significantly enriched (*p* < 0.05) after PBZ treatment. Protein processing in endoplasmic reticulum (ko04141), circadian rhythm-plant (ko04712), flavonoid biosynthesis (ko00941), and diterpenoid biosynthesis (ko00904) were significantly enriched among the three PBZ-treated samples, while spliceosome (ko03040) and phosphatidylinositol signaling system (ko04070) were significantly enriched in T3 sample and T72 sample. In addition, specific enrichment was observed for endocytosis (ko04144) in T3 sample, plant-pathogen interaction (ko04626) in T24 sample and phenylpropanoid biosynthesis (ko00940) in T72 sample (Table 3). Generally, Spliceosome, protein processing in endoplasmic reticulum and endocytosis were involved in gene expression, protein synthesis and transportation regulation, respectively. Diterpenoid biosynthesis, flavonoid biosynthesis and phenylpropanoid biosynthesis were involved in GA signal and secondary metabolites which played important role in plant growth and antioxidant activity. Circadian rhythm-plant, phosphatidylinositol signaling system and plant-pathogen interaction regulated signaling transduction and environmental response.

**Table 3:** **List of the enriched KEGG pathways compared with control group**.

**Pathway ID**	**KEGG pathway**	**DEGs with pathway annotation**			**All genes with pathway**
		**T3 vs. CK (471)**	**T24 vs. CK (234)**	**T72 vs. CK (462)**	**annotation (25092)**
ko04141	Protein processing in endoplasmic reticulum	93(19.6%)^**^	31(13.5%)^**^	84(18.2%)^**^	677(2.7%)
ko04712	Circadian rhythm-plant	12 (2.6%)^**^	12 (5.2%)^**^	23(5.0%)^**^	229 (0.9%)
ko00904	Diterpenoid biosynthesis	9 (1.9%)^**^	3(1.3%)^*^	6(1.3%)^**^	80 (0.3%)
ko00941	Flavonoid biosynthesis	19 (4.0%)^**^	9 (3.8%)^**^	14 (3.0%)^**^	241 (1.0%)
ko03040	Spliceosome	32 (6.8%)^**^	14 (6.1%)	33 (3.0%)^**^	1055(4.2%)
ko04070	Phosphatidylinositol signaling system	7 (1.5%)^*^	4 (1.7%)	9(2.0%)^**^	172(0.7%)
ko04144	Endocytosis	76 (16.1%)^**^	27 (11.7%)	61(13.2%)	3038 (12.1%)
ko04626	Plant-pathogen interaction	16(3.4%)	24 (10.4%)^**^	21 (4.6%)	1108 (4.4%)
ko00940	Phenylpropanoid biosynthesis	9 (1.9%)	4 (1.7%)	14 (3.0%)^*^	413 (1.7%)

* Indicated the significant difference (p < 0.05);

** *Indicated the very significant difference (p < 0.01)*.

### DEGs involved in cell division and cell expansion

**Figure 8:**
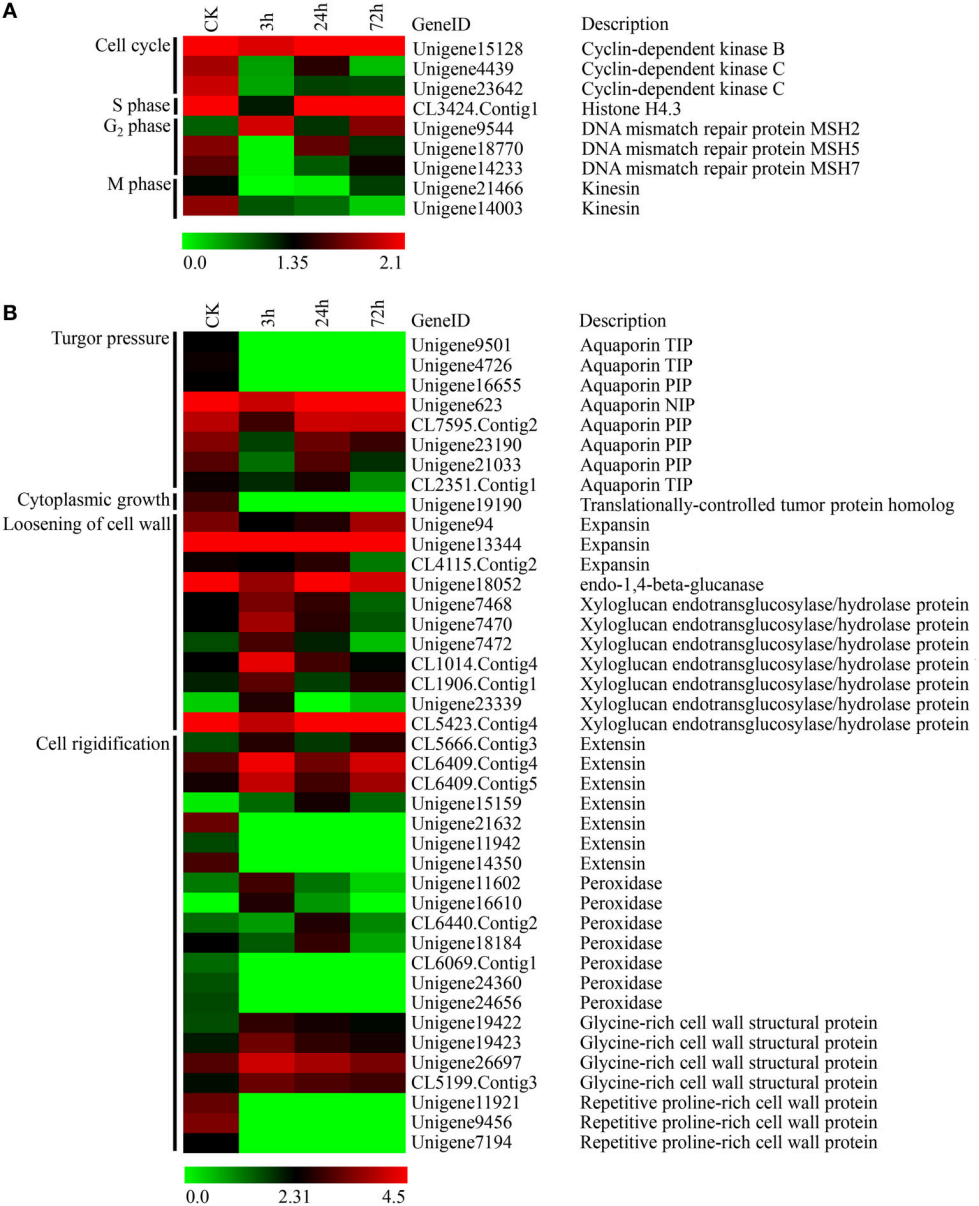
**Expression profiles of DEGs involved in cell division and cell expansion in lily leaves by PBZ treatment. (A)** Genes involved in cell division. **(B)** Genes involved in cell expansion. The bar represents the scale of the expression levels for each gene (log_2_FPKM) in the CK, PBZ-treated sample at 3, 24 and 72 h. Red rectangles indicate upregulation of genes, and green rectangles indicate down-regulation. Complete information for each gene list can be found in Table S4.

In this study, we observed that PBZ significantly reduced leaf size in *L*. LA hybrid. Leaf size is governed by cell division and cell expansion processes. Thus, the DEGs associated with cell division and cell expansion were screened. Cell division is highly regulated by sequential progression through cell cycle phases. According to the phases of the cell cycle, nine DEGs associated with cell division included three genes involved in the cell cycle (encoding CDKs), one gene involved in S phase (encoding histone H4.3), three genes involved in G2 phase (encoding DNA mismatch repair protein MSH2/MSH5/MSH7), and two genes involved in M phase (encoding kinesin) (Figure 8A). In these results, the expression levels of these genes all declined except the *MSH2* gene after PBZ treatment, especially at 3 h (Figure 8A).

On the basis of the cell expansion process, 41 DEGs associated with cell growth were classified into one gene involved in cytoplasmic growth (encoding translationally-controlled tumor protein homolog TCTP), eight genes involved in turgor pressure (encoding aquaporin NIP/PIP/TIP), 11 genes involved in cell wall loosening (encoding expansins, endo-1,4-beta-glucanase and XTHs) and 21 genes involved in cell wall rigidification (encoding extensins, glycine-rich cell wall structural proteins, proline-rich cell wall proteins and peroxidases) (Figure 8B). The results showed that the expression levels of genes encoding aquaporins (AQPs), TCTP, expansin and endo-1, 4-beta-glucanase appeared to decline after PBZ treatment (Figure 8B). However, there were complicated expression patterns for three other groups of genes, namely those encoding XTHs, extensins and peroxidases. Some of genes encoding homologous products were upregulated, but others were downregulated by PBZ (Figure 8B).

### DEGs associated with GA metabolism and signal transduction

The studies in other species showed that PBZ interfered with GA metabolism. Here, we also found that diterpenoid biosynthesis pathway including GA metabolism was significantly enriched after PBZ treatment (Table 3). Thus, the DEGs involved in the entire GA metabolism and signaling transduction were focused.

**Figure 9:**
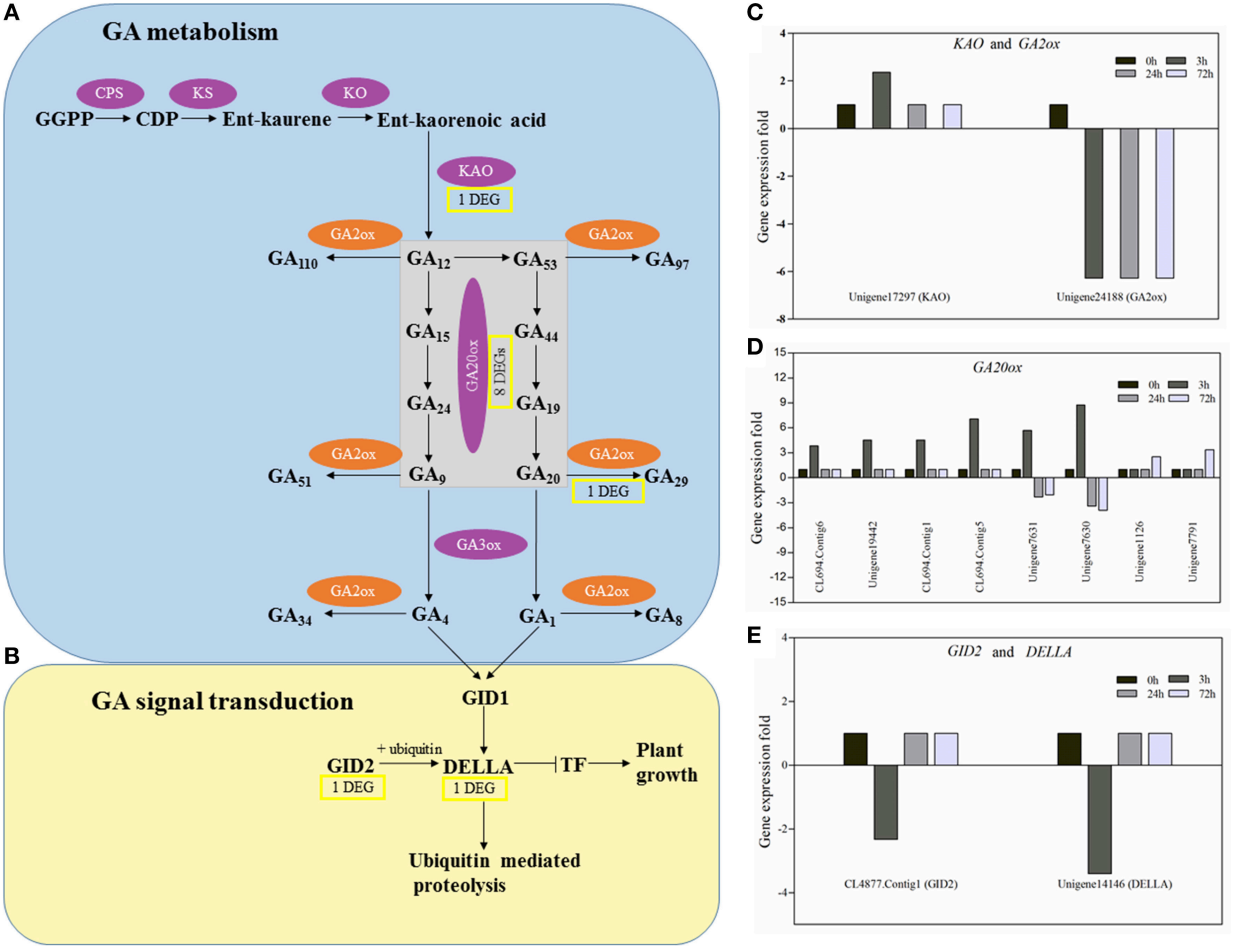
**The principal pathways of GA metabolism and signal transduction and the expression profile of DEGs involved in GA metabolism and signal transduction. (A)** GA metabolism. **(B)** GA signal transduction. GA biosynthetic enzymes are indicated in the purple ovals, and GA metabolic enzymes in the orange ovals. Yellow rectangles represent the numbers of DEGs encoding enzymes or components of GA metabolism and signal transduction in PBZ-treated lily leaves. The arrows represent catalytic reaction, 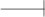 represents inhibition. **(C–E)** The histograms showed expression profiles of these DEGs in the CK, PBZ-treated sample at 3, 24, and 72 h.

GA biosynthesis can be separated into the early stage involved in terpene cyclases (CPS and KS) and monooxygenases (KO and KAO) and the late stage involved in dioxygenases (GA20ox and GA3ox; Figure 9A). Our results showed that in the early stage of GA biosynthesis, a *KAO* gene (unigene17297) slightly and temporarily increased and then rapidly returned to a normal level (Figure 9C). In the late stage of GA biosynthesis, eight *GA20ox* genes were found in response to PBZ. Based on their expression pattern, they could be grouped into three classes. Genes in the first (CL694.Contig6, unigene19442, CL694.Contig1, CL694.Contig5) and second (unigene7630, unigene7631) classes were all affected in the early stage of PBZ treatment (3 h) but differed in their expression patterns. Genes in the first class were quickly upregulated and then returned to a normal level, whereas those in the second class were quickly upregulated at 3 h and then downregulated at 24 and 72 h. Genes in the third class (unigene1126, unigene7791) were affected in the late stage of PBZ treatment (72 h) and were upregulated (Figure 9D). In the GA deactivation, the transcript of a *GA2ox* gene (unigene24188) decreased quickly and continuously after PBZ treatment (Figure 9C). In the GA signaling transduction (Figure 9B), one *GID2* gene (CL4877.contig1) and one *DELLA* gene (unigene14146) were found in response to PBZ and they had a similar expression profile, where they were rapidly downregulated at 3 h but returned to normal at 24 and 72 h (Figure 9E).

## Discussion

So far, there is no genomic data for *Lilium* plants. Thus, the RNA-seq is the most powerful and attractive tool for in-depth analysis at molecular level. In the present study, we performed de novo assembly of *L*. L.A. hybrid used RNA-seq technology. Approximately 38.6 Gb of data was generated and assembled into 53,681 unigenes, of which 38,507 were annotated in Nr, Nt, Swiss-Prot, KEGG, COG, and GO databases. These data will be valuable to build genomic resources for genetic studies in lily. Plant height control is one object of lily breeding, and a common and effective means is PGRs application at present. Here, this was the first study to compare global gene expression profiles after PBZ treatment in lily by RNA-seq technology. 2704 DEGs were revealed and 9 metabolic pathways and signal transduction pathways were significantly enriched. According to these results, we tried to understand comprehensively how PBZ impacted on growth and development of lily and further investigated into the molecular mechanism of lily dwarfism, which could be applied to research on molecular breeding of plant type in lily. Due to transcriptional response prior to phenotypic expression, we focused on the three earlier time points following PBZ treatment to seek out PBZ-responsive genes, including early responsive and late responsive genes, as fully as possible.

Transcriptome analysis could provide a comprehensive understanding on biological function of genes. In this study, nine metabolic pathways and signaling transduction pathways were enriched in response to PBZ. These pathways involved in a wide range of molecular processes from gene expression to protein, hormone signal and secondary metabolites synthesis, and from signaling transduction to transportation regulation. Thus, it was speculated that a variety of molecular processes in response to PBZ may lead to different morphological and physiological responses in leaves including reducing leaf area (Nair et al., 2009; Cohen et al., 2013), increasing chlorophyll contents (Sunitha et al., 2004), improving level of antioxidants (Srivastav et al., 2010) and enhancing resistance to abiotic stresses (Ozmen et al., 2003; Zhu et al., 2004; Lin et al., 2006) and so on. For example, flavonoid had antioxidant activity and resistance to adverse environment such as UV light. In our results, PBZ affected expression level of unigenes encoded key enzymes in the flavonoid biosynthesis including phenylalanine ammonia-lyase, chalcone synthase, flavonol synthase, flavonoid 3-hydroxylase, naringenin 3-dioxygenase, flavanone 3-dioxygenase, and anthocyanidin synthase (Table S5) at molecular level, which could partly explain that PBZ improved antioxidants and enhanced resistance to abiotic stresses.

However, the most significant impact of PBZ is inhibiting stem and leaves growth. In this study, we also found that leaf length in *L*. LA hybrid was significantly decreased by PBZ and further observation at cellular level showed cell length also was reduced (Figure 2). Leaf size relies on the precise regulation of cell number and/or size, which are governed by cell division and cell expansion processes (González and Inzé, 2015). Mutation or expression alteration of genes involved in these processes was related to dwarfism. Our results showed that the expression pattern of many cell division and cell expansion-related genes significantly changed in response to PBZ.

In the cell division process, the expression levels of three genes encoding CDKs and two genes encoding kinesins decreased. CDKs govern the plant cell cycle through triggering G1-to-S and G2-to-M transition (Inzé and De Veylder, 2006). Yoshizumi et al. (Yoshizumi et al., 1999) reported that inhibition of the expression of the *cdc2b* gene encoding B-type CDK resulted in short-hypocotyl and open-cotyledon phenotypes in *Arabidopsis*. Kinesin family proteins are microtubule-based motor proteins, which are involved in cell division and elongation in plants (Li et al., 2012). Mutation of *OsNACK1*, a kinesin family member, leads to severe dwarfism in rice (Sazuka et al., 2005). In addition, the expression pattern of one gene (*Histone H4.3*) involved in DNA synthesis and three genes (*MSH2, MSH5*, and *MSH7*) involved in DNA mismatch repair system were also affected by PBZ, and their expression levels all declined except the *MSH2* gene. These results indicated that suppressed expression of these genes probably helped to dwarfism through regulating cell division process after PBZ treatment in lily.

In contrast to cell division, the expression pattern of more genes involved in the cell expansion changed in response to PBZ. Our results showed that the expression levels of genes encoding AQPs, TCTP, expansins and endo-1, 4-beta-glucanase appeared to decline while cell size decreased after PBZ treatment. Especially for the expansin gene, Azeez also demonstrated that the gladiolus expansin gene *GgEXPA1* was inhibited by PBZ (Azeez et al., 2010). Function researches in other species have shown that the down-expression or silence of these homologous genes lead to slow growth or dwarfism, but their overexpression accelerate plant growth. Cell expansion in leaves requires the continuous uptake of water through water channels, called AQPs, which are probably involved in leaf growth by modifying membrane permeability (Heinen et al., 2009). In tobacco, overexpression of *NtAQP1* gene increases leaf growth rate (Uehlein et al., 2003). TCTP has been shown to play a role in the regulation of cell growth and the control of the cell cycle in yeast and human cell lines (Yarm, 2002). Berkowitz demonstrated that *TCTP* gene silencing inhibited leaf expansion due to reduced cell size in *Arabidopsis* (Berkowitz et al., 2008). Expansins are thought to bind to cellulose and to disrupt the noncovalent bonds between cellulose and matrix polysaccharides to induce cell wall loosening (Mcqueenmason and Cosgrove, 1995). In *Petunia* hybrid, downregulation of an α-expansin gene, *PhEXP1*, was shown to reduce epidermal cell area in petal size (Zenoni et al., 2004). By contrast, overexpression of expansin genes has been reported to increase growth (Choi et al., 2003). However, there were complicated expression patterns for three groups including genes encoding XTHs, extensins and peroxidases in response to PBZ. Some genes were upregulated, but others were downregulated by PBZ (Figure 8B). XTHs play an important role in restructuring cell wall, and comprehensive expression analysis of *XTH* genes in *Arabidopsis* shows that they respond differently to various environmental cues and hormonal signals such as elevated expression of the *XTH23* gene and suppression of *XTH26* gene expression by GA (Yokoyama and Nishitani, 2001). One possible explanation for distinct expression patterns responding to the same signal is the versatile functions of XTHs, since it has been demonstrated that the overexpression of some *XTH* genes in plants promote growth (Shin et al., 2006; Nishikubo et al., 2011), while others have no effect (Verbelen and Vissenberg, 2007; Miedes et al., 2011). There is also evidence that XTHs play a role in both wall loosening and strengthening (Antosiewicz et al., 1997). Extensins and peroxidases are responsible for forming cross-links between cell wall polymers and proteins to strengthen the cell wall and stop cell expansion (Borner et al., 2002; Showalter et al., 2010). Like *XTH* genes, the homologous genes encoding extensins or peroxidases also comprise a large family and play various roles in the whole life cycle of a plant (Passardi et al., 2004; Showalter et al., 2010), and thus they also probably have different responses to the same signal. It was speculated that these cell expansion-related genes were regulated by PBZ and contributed to lily dwarfism.

It is reported that cell expansion and cell division processes are controlled by GA, which is the important phytohormone regulating a variety of developmental processes, including stem and root elongation, seed germination, floral development, and determination of leaf size and shape (Ubeda-Tomás et al., 2008; Achard et al., 2009). The mechanism study of PBZ also reveals that it lead to short stem and reduced leaves through decreasing GA content in plants (Hedden and Graebe, 1985). In our study, the diterpenoid biosynthesis pathway was significantly enriched, and by further analyzing this pathway, expression of genes only involved in GA metabolism changed in response to PBZ (Images 1–3). Nine genes encoding enzymes involved in GA biosynthesis (one *KAO* genes and eight *GA20ox* genes) were upregulated and one gene encoding enzymes involved in GA deactivation (*GA2ox* gene) was downregulated by PBZ. These results were consistent with other studies that reduction of bioactive GA levels usually upregulate the expression of *GA20ox* genes (Phillips et al., 1995) and downregulate *GA2ox* genes (Thomas et al., 1999). This phenomenon was explained by GA feedback regulation to sustain GA homeostasis. But the difference was that a *KAO* gene slightly and temporarily increased (Figure 9C). there is some evidence that none of genes in the early steps of the GA biosynthetic pathway including *KAO* appears to show feedback regulation in *Arabidopsis* and pea (Silverstone et al., 1997; Helliwell et al., 1998; Davidson et al., 2005). This is probably because the *KAO* gene may be an early responsive and transiently induced gene in response to PBZ, but its expression change within 3 h after PBZ treatment was seldom reported in previous studies. Another a possibility was the difference between lily and other species. Above all, when PBZ is used to spray the lily seedlings, the feedback mechanism of GA metabolism is initiated due to low GA content.

Like GA metabolic pathways, some components of GA signaling transduction are also regulated by the feedback mechanisms of GA levels. The DELLA proteins act as repressors of GA signaling and are destabilized by GA (Ikeda et al., 2001). The expression levels of genes encoding DELLA proteins were upregulated in response to GA_3_ in *Arabidopsis* and rice shoots (Silverstone et al., 1998; Ogawa et al., 2000) and downregulated in gibberellin-deficient plant (*ga1-3*) and *GA2ox1* overexpression plant (*GA2ox1OE*) (Middleton et al., 2012). In this study, one *DELLA* gene was shortly downregulated in response to PBZ (Figure 9E). Middleton (Middleton et al., 2012) reported that a decrease in GA levels led to an increase in DELLA protein concentrations due to gibberellin-mediated degradation, and that a high DELLA protein concentration repressed *DELLA* transcription. GID2, an F-box protein, forms an SCF^GID2^ complex that acts as an SCF E3 ubiquitin ligase targeting DELLA (Sasaki et al., 2003). In this study, one *GID2* gene had a similar expression profile to *DELLA* gene in response to PBZ (Figure 9E). In conclusion, PBZ also initiates feedback mechanism of GA signaling transduction to rescue GA homeostasis.

Genetic analysis in other species indicated that modulation of genes related to GA metabolism and signaling transduction pathway had close relationships with plant dwarfism (Hedden and Phillips, 2000; Olszewski et al., 2002). Mutation of *GA20ox* gene in rice exhibited semi-dwarf trait combined with improved crop yield (Monna et al., 2002; Sasaki et al., 2002). Overexpression of *GA2ox* gene enhanced gibberellin inactivation and induced dwarfism in *Solanum* species (Dijkstra et al., 2008). In wheat, mutation of *Rht1* gene encoding DELLA protein led to reduced height and produced “green revolution wheat” (Peng et al., 1999). Therefore, these homologous genes in lily are promising targets for genetic breeding for short lily.

## Conclusions

Paclobutrazol is an efficient plant growth retardant that is commonly used in potted plants. With the development of genetic engineering breeding technology, it is expected to be an alternative to the use of plant growth retardant. This study revealed that the cellular process (cell division and cell expansion) and the GA signal (GA metabolism and GA signaling transduction) responded to PBZ at the transcript level by RNA-seq. Some genes potentially related to dwarfism were highlighted to reveal their importance in regulation of plant size. The main contribution of the study is to lay foundation for dwarf breeding by genetic engineer to promote the development of potted lilies.

## Author contributions

LS was responsible for research costs and guided the work design and manuscript writing. XZ and MC contributed equally to this work including designing the work, conducting the work, data analysis, writing manuscript and revising manuscript. YL took part in designing the work, data analysis and revising manuscript. MS took part in data analysis and revising manuscript. JZ, GS, and CJ took part in designing the work and revising manuscript, provides materials source and guides cultivation of material. All the authors approved the final version of the submitted manuscript.

### Conflict of interest statement

The authors declare that the research was conducted in the absence of any commercial or financial relationships that could be construed as a potential conflict of interest.

## Supplementary material

Table S1 **The primer sequences of 12 unigenes for qRT-PCR**.

Table S2 **Output statistics of transcriptome sequencing**.

Table S3 **The enriched 128 KEGG pathways**.

Table S4 **Complete information for gene list in Figure 8**.

Table S5 **List of DEGs involved in flavonoid biosynthesis**.

Image 1 **Diterpenoid biosynthesis pathway in KEGG after PBZ-treatment 3 h**. Rectangles with numbers display enzymes. Small circles represent compounds. Green rectangles represent downregulated genes and red rectangles represent upregulated genes in lily leaves after PBZ treatment.

Image 2 **Diterpenoid biosynthesis pathway in KEGG after PBZ-treatment 24 h**. Rectangles with numbers display enzymes. Small circles represent compounds. Green rectangles represent downregulated genes in lily leaves after PBZ treatment.

Image 3 **Diterpenoid biosynthesis pathway in KEGG after PBZ-treatment 72 h**. Rectangles with numbers display enzymes. Small circles represent compounds. Green rectangles represent downregulated genes and red rectangles represent upregulated genes in lily leaves after PBZ treatment.
