# Supplementary material for: Global Transcriptome Profiling Analysis of Inhibitory Effects of Paclobutrazol on Leaf Growth in Lily (Lilium Longiflorum-Asiatic Hybrid)
Source: Front Plant Sci. 2016 Apr 19;7:491. doi: 10.3389/fpls.2016.00491 (PMC4835717; doi:10.3389/fpls.2016.00491)
Supplement: Table S1 — The primer sequences of 12 unigenes for qRT-PCR. [file Table1.DOC]

**Table S1 The primer sequences of 12 unigenes for qRT-PCR**

| **Gene ID** | **Primer name** | **Sequence(5'to 3')** | **Sequence length (bp)** |
| --- | --- | --- | --- |
| Unigene17297 | Unigene17297-Forward | GACTCCTTCCTCGCCTCCTT | 150 |
|  | Unigene17297-Reverse | CGGCCAACCAGGTACAAATC |  |
| Unigene7630 | Unigene7630-Forward | TTGCTGTTCTTCCGGGTCTT | 210 |
|  | Unigene7630-Reverse | CATCCAGGTGTGGAGCAATG |  |
| Unigene7631 | Unigene7631-Forward | TTGCTGTTCTTCCGAGTCTTCA | 210 |
|  | Unigene7631-Reverse | CATCCAGGTGTGGAGCAATG |  |
| Unigene18845 | Unigene18845-Forward | AGGCTTGGAGCAATGATGTCT | 140 |
|  | Unigene18845-Reverse | GTACAGTGAAGGCTCCATGCA |  |
| Unigene14146 | Unigene14146-Forward | TATGCTGCTACCTTCGATTCTTTG | 220 |
|  | Unigene14146-Reverse | TGCTTTGAATGATTTCCCTCTCT |  |
| Unigene15128 | Unigene15128-Forward | GAGTCGCCCCAAGAAGCA | 251 |
|  | Unigene15128-Reverse | GGAGACGACTCCGAAGAATGC |  |
| Unigene94 | Unigene94-Forward | CCATTGCTCTGCCAGTTCTG | 180 |
|  | Unigene94-Reverse | GGTGCCGTGCGTGAAGA |  |
| Unigene7468 | Unigene7468-Forward | AGAGGATGGAGTAGGTGTGGAAGT | 160 |
|  | Unigene7468-Reverse | CCCACCCACGACGAGATC |  |
| CL2975.Contig1 | CL2975.Contig1-Forward | GTCCCTGTGGTGCTCATTGA | 215 |
|  | CL2975.Contig1-Reverse | TTGGTTTTCTTCCCCTTGGA |  |
| CL838.Contig2 | CL838.Contig2-Forward | GTGAAGGCCGAAGACAAAGC | 165 |
|  | CL838.Contig2-Reverse | TAATTGGTTGCGGGCATCA |  |
| Unigene9783 | Unigene9783-Forward | CCCTGGTCCTTGTCCTTTCA | 165 |
|  | Unigene9783-Reverse | CCCCTCTCTCCCTCGGTCTA |  |
| CL3392.Contig1 | CL3392.Contig1-Forward | CGGACGCCTACCTCTACTTCA | 215 |
|  | CL3392.Contig1-Reverse | CTTCCACACCGCCGAGACT |  |
