# Supplementary material for: Global Transcriptome Profiling Analysis of Inhibitory Effects of Paclobutrazol on Leaf Growth in Lily (Lilium Longiflorum-Asiatic Hybrid)
Source: Front Plant Sci. 2016 Apr 19;7:491. doi: 10.3389/fpls.2016.00491 (PMC4835717; doi:10.3389/fpls.2016.00491)
Supplement: Table S2 — Output statistics of transcriptome sequencing. [file Table2.DOC]

**Table S2 Output statistics of transcriptome sequencing**

| **Samples** | **Q20 percentage** | **GC percentage** | **Number of contigs** | **Mean length of contigs (nt)** |
| --- | --- | --- | --- | --- |
| CK-1 | 97.31% | 52.66% | 96452 | 308 |
| CK-2 | 97.39% | 52.43% | 92896 | 312 |
| T3-1 | 97.48% | 52.48% | 94064 | 311 |
| T3-2 | 97.47% | 52.77% | 93351 | 315 |
| T24-1 | 97.47% | 52.64% | 90995 | 321 |
| T24-2 | 97.50% | 52.58% | 97050 | 315 |
| T72-1 | 97.54% | 52.16% | 95462 | 314 |
| T72-2 | 96.60% | 52.09% | 96357 | 315 |
