# Supplementary material for: Global Transcriptome Profiling Analysis of Inhibitory Effects of Paclobutrazol on Leaf Growth in Lily (Lilium Longiflorum-Asiatic Hybrid)
Source: Front Plant Sci. 2016 Apr 19;7:491. doi: 10.3389/fpls.2016.00491 (PMC4835717; doi:10.3389/fpls.2016.00491)
Supplement: Table S5 — List of DEGs involved in flavonoid biosynthesis. [file Table5.DOC]

**Table S5 List of DEGs involved in flavonoid biosynthesis**

| **Description** | **GeneID** | **Log_2_ratio**  **(3h/CK)** | **Log_2_ratio**  **(24h/CK)** | **Log_2_ratio**  **(72h/CK)** |
| --- | --- | --- | --- | --- |
| Phenylalanine ammonia-lyase | CL8524.Contig1 | 0.66 | 0.43 | 1.15 |
|  | CL8524.Contig4 | 0.68 | 0.32 | 1.09 |
|  | CL8524.Contig6 | 0.63 | 0.38 | 1.35 |
| Agmatine coumaroyltransferase | CL4551.Contig4 | 0.60 | 0.08 | 1.10 |
|  | Unigene17940 | -1.90 | -0.29 | -1.90 |
|  | CL26.Contig3 | -1.10 | -0.31 | -0.81 |
| Chalcone synthase | Unigene21513 | 2.28 | -1.22 | -0.64 |
|  | Unigene18388 | 2.45 | -0.83 | -0.38 |
|  | CL5932.Contig2 | 0.58 | 0.39 | 1.03 |
|  | CL844.Contig6 | 0.57 | 0.81 | 1.42 |
|  | CL844.Contig7 | 0.61 | 0.51 | 1.10 |
|  | Unigene13149 | 0.53 | 0.72 | 1.02 |
|  | CL6148.Contig1 | -3.11 | -3.11 | -3.11 |
|  | CL6148.Contig2 | -3.18 | -3.18 | -3.18 |
|  | CL8508.Contig2 | -2.29 | -2.29 | -2.29 |
|  | CL8508.Contig3 | -2.20 | -2.20 | -2.20 |
|  | CL8508.Contig1 | -3.14 | -3.14 | -3.14 |
| Naringenin 3-dioxygenase | CL327.Contig1 | 0.04 | -0.87 | -1.12 |
|  | CL327.Contig2 | -0.30 | -0.88 | -1.31 |
|  | CL4803.Contig1 | 1.37 | -0.32 | -0.13 |
| Flavanone 3-dioxygenase | CL9516.Contig1 | 1.81 | -1.24 | 0.41 |
|  | CL9516.Contig2 | 2.38 | -1.08 | -0.30 |
|  | CL9516.Contig3 | 1.94 | -1.08 | -0.52 |
|  | CL9516.Contig4 | 1.39 | -0.48 | -0.05 |
| Flavonol synthase/flavonoid 3-hydroxylase | CL694.Contig2 | 2.54 | -0.54 | 0.01 |
|  | CL694.Contig4 | 2.37 | -0.30 | 0.00 |
|  | CL694.Contig5 | 2.82 | -0.91 | 0.15 |
|  | CL694.Contig1 | 2.18 | -0.96 | -0.84 |
| Anthocyanidin synthase | Unigene25280 | 1.64 | 0.34 | 0.28 |
| Isoflavone reductase | Unigene3570 | 0.92 | -0.40 | -1.17 |

Note: red numbers represent that gene expression elevated more than two folds compared with control group. Green numbers represent that gene expression declined more than two folds compared with control group.
