# Supplementary figures and images for: Global Transcriptome Profiling Analysis of Inhibitory Effects of Paclobutrazol on Leaf Growth in Lily (Lilium Longiflorum-Asiatic Hybrid)
Source: Front Plant Sci. 2016 Apr 19;7:491. doi: 10.3389/fpls.2016.00491 (PMC4835717; doi:10.3389/fpls.2016.00491)

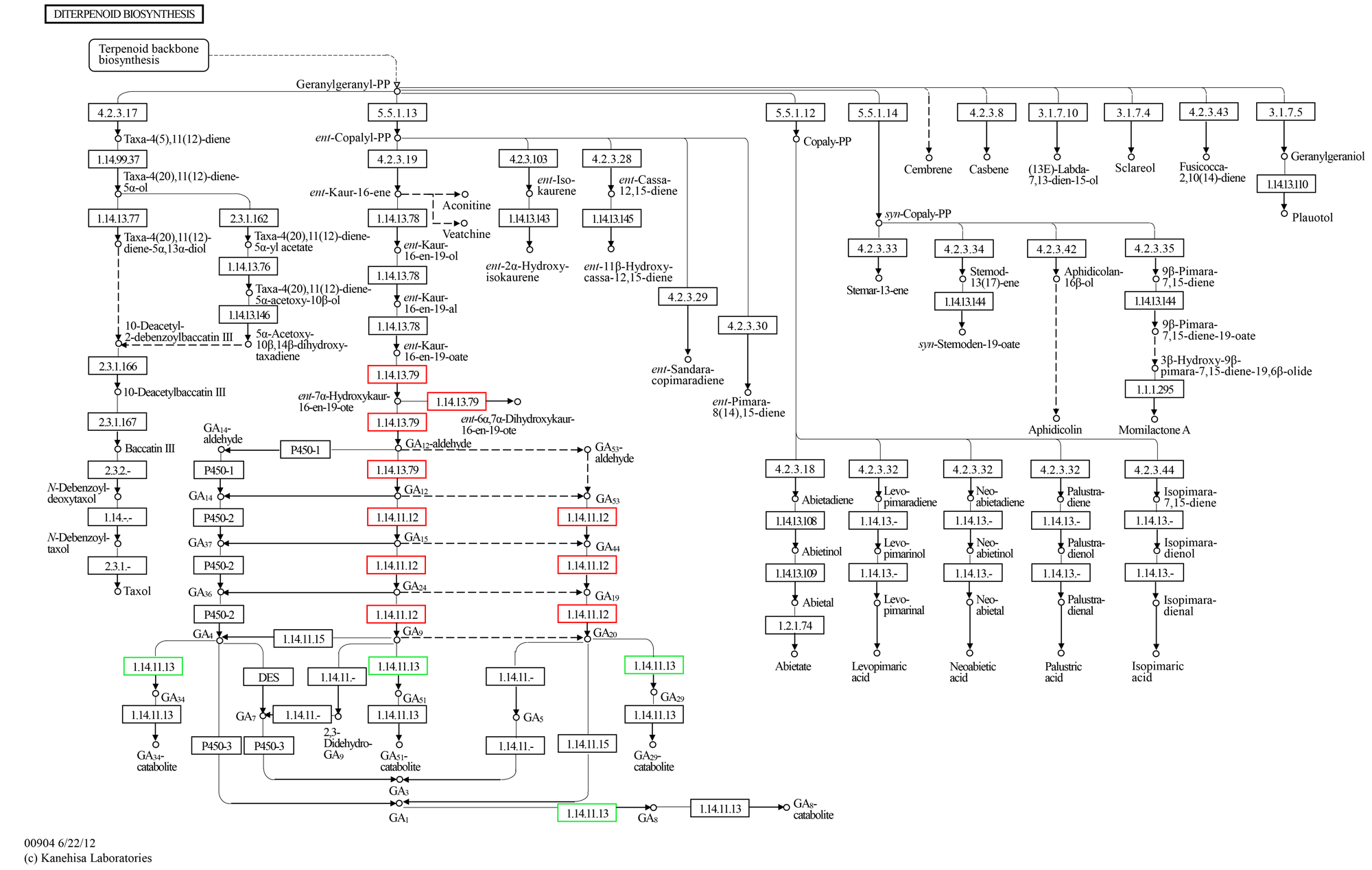

Supplement: Image 1 — Diterpenoid biosynthesis pathway in KEGG after PBZ-treatment 3 h. Rectangles with numbers display enzymes. Small circles represent compounds. Green rectangles represent downregulated genes and red rectangles represent upregulated genes in lily leaves after PBZ treatment. [file Image1.TIF]

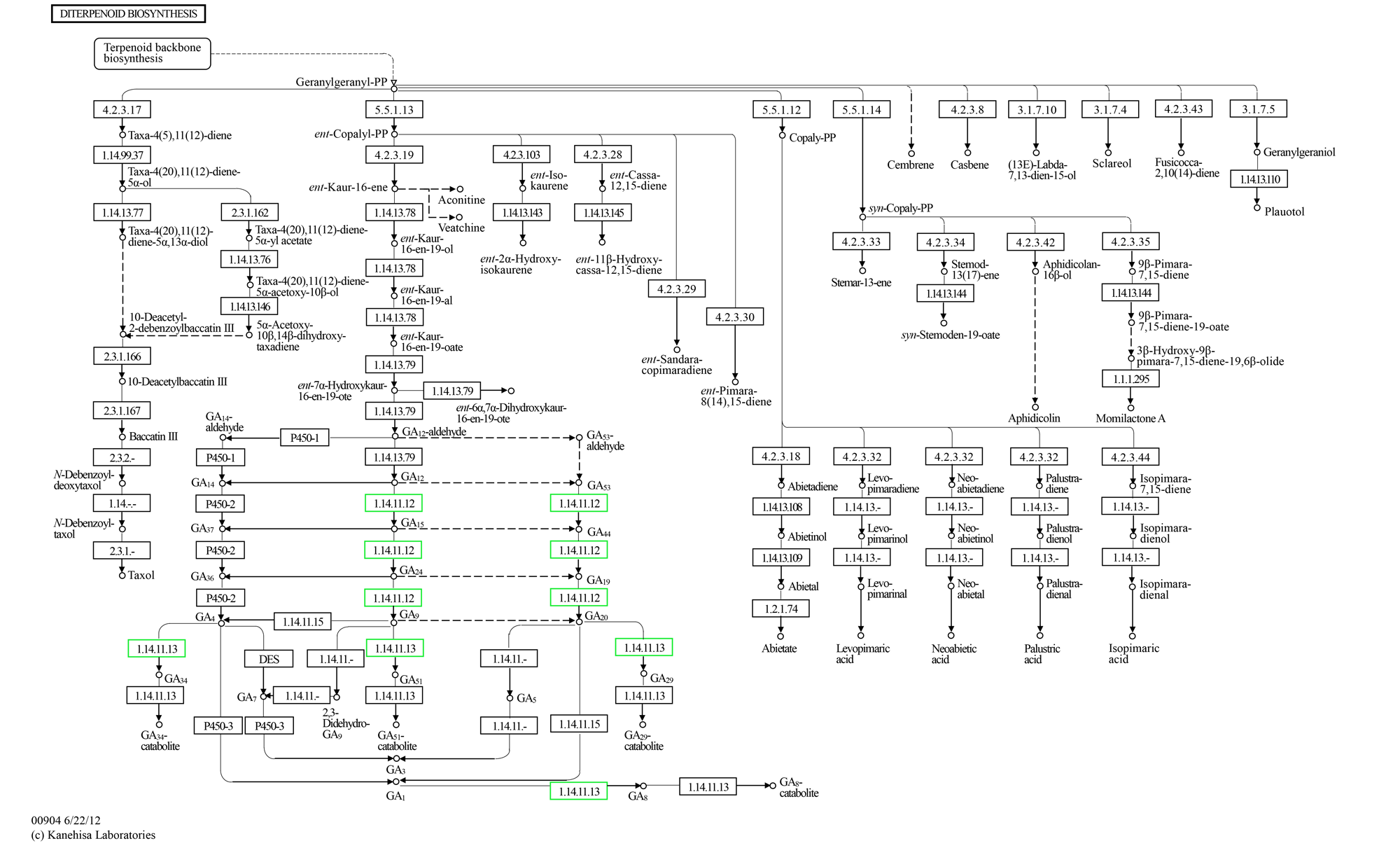

Supplement: Image 2 — Diterpenoid biosynthesis pathway in KEGG after PBZ-treatment 24 h. Rectangles with numbers display enzymes. Small circles represent compounds. Green rectangles represent downregulated genes in lily leaves after PBZ treatment. [file Image2.TIF]

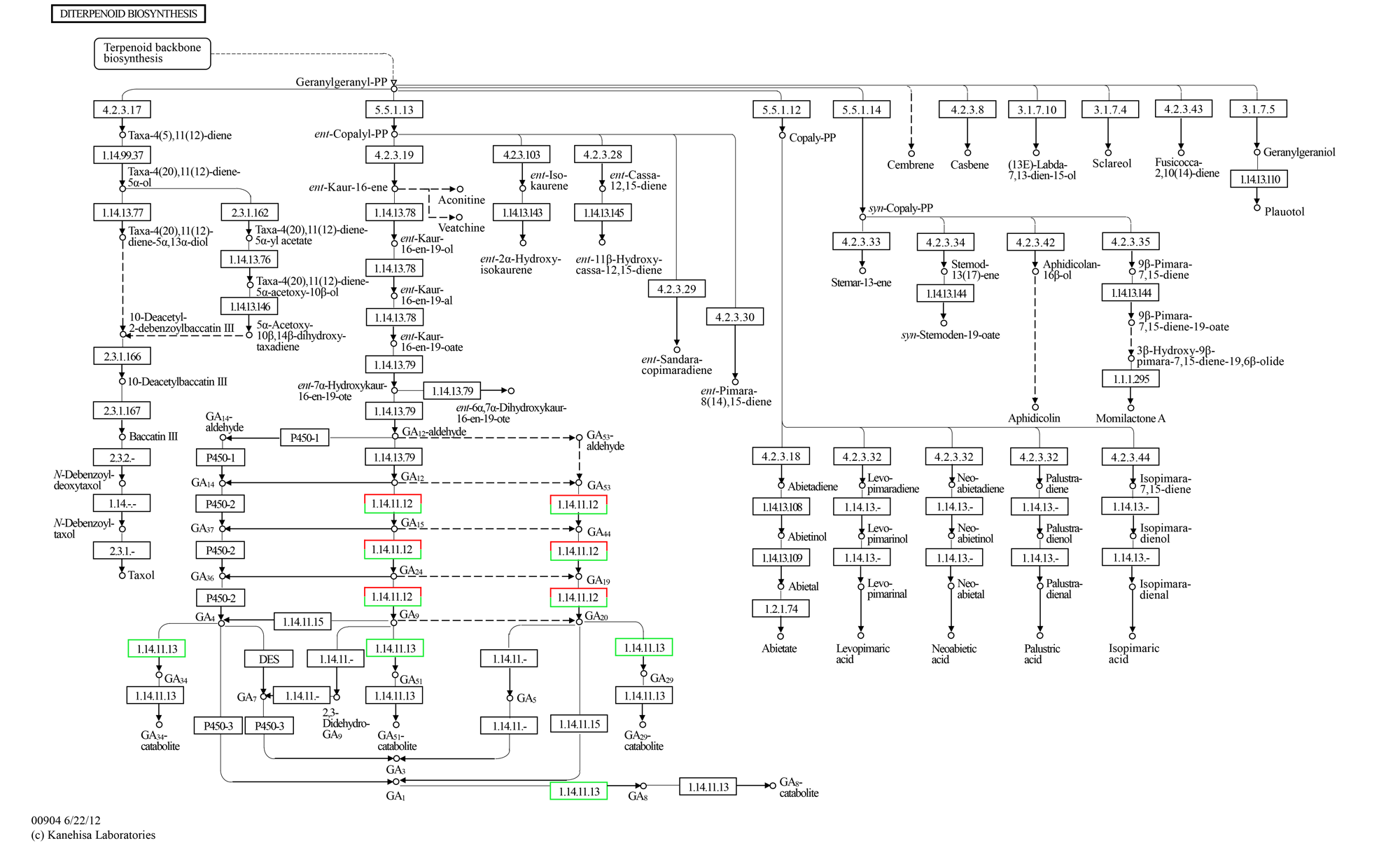

Supplement: Image 3 — Diterpenoid biosynthesis pathway in KEGG after PBZ-treatment 72 h. Rectangles with numbers display enzymes. Small circles represent compounds. Green rectangles represent downregulated genes and red rectangles represent upregulated genes in lily leaves after PBZ treatment. [file Image3.TIF]
